# Background levels of polycyclic aromatic hydrocarbons and legacy organochlorine pesticides in wheat sampled in 2017 and 2018 in Poland

**DOI:** 10.1007/s10661-020-8097-5

**Published:** 2020-01-25

**Authors:** Marek Łukasz Roszko, Karolina Juszczyk, Magdalena Szczepańska, Olga Świder, Krystyna Szymczyk

**Affiliations:** Department of Food Analysis, Institute of Agricultural and Food Biotechnology, Rakowiecka 36, 02-532 Warsaw, Poland

**Keywords:** Wheat, Cereals, PAH, Insecticides, DDT, Risk assessment

## Abstract

Both polycyclic aromatic hydrocarbons (PAHs) and legacy organochlorine insecticides (OCPs), including DDT, are dangerous chemical contaminants. The aims of this study were to (i) determine background levels of PAHs and legacy OCPs for wheat samples collected in 2017 and 2018 in Poland, (ii) identify differences between levels in wheat harvested in various regions of Poland, (iii) evaluate differences in contamination sources manifested by the profiles of the identified chemicals, (iv) identify possible correlations between different classes of chemicals present in wheat, and (v) assess the health risks associated with the presence of PAHs and OCPs in Polish wheat. Average concentrations found in the samples were 0.09 ± 0.03 μg kg^−1^ for benzo[a]pyrene (BaP) (formerly used as a single PAH marker), 0.43 ± 0.16 for the more recently introduced collective PAH 4 marker (benzo[a]anthracene + benzo[a]pyrene + chrysene + benzo[b]fluoranthene), and 1.07 ± 0.68 μg kg^−1^ for DDT and its metabolites. The PAH profiles indicated contamination from combustion-related emission sources (liquid fossil fuels, coal, biomass). Health risks associated with the presence of PAHs and OCPs in cereals were assessed using the margin of exposure (MOE) approach. The MOE values calculated based on the highest concentrations found in this study exceeded 50,000 for both BaP and PAH 4. The calculated worst-case scenario value for DDT and metabolites was as low as 0.3% of the respective tolerable daily intake (TDI) value. Assessment of dietary risk has shown that the presence of the two contaminant classes in Polish wheat grains is of low concern.

## Introduction

A vast number of organic pollutants found in unprocessed cereal grains can be generally divided into substances of biological origin and residues of various chemicals. Mycotoxins produced by molds are a common example of the former (Bryła et al. 2019). Chemical pollutants primarily include pesticides purposefully used by farmers to protect their crops against various pests/weeds (Jagadish et al. 2015) as well as chemicals that migrate from the environmental background, such as dioxins, chlorinated biphenyls, DDT and other legacy crop protection substances, polybrominated diphenyl ethers, or polycyclic aromatic hydrocarbons (PAHs) (Roszko and Szymczyk 2009; Roszko et al. 2014, 2016).

PAH molecules consist of a number of condensed aromatic rings that form selected planar structures and are produced as a result of incomplete combustion of carbon-containing materials, including organic matter (Lawal 2017). PAHs are also found in crude oil, and thus, oil extraction/production might also be responsible for the presence of petrogenic PAHs in environmental samples (Lawal 2017; Pampanin and Sydnes 2017). PAHs are highly lipophilic, with octanol/water partitioning coefficients (expressed as log o/w) exceeding 3, and are chemically moderately inert (Pinsuwan et al. 1995).

The PAHs in various environmental/wildlife/food samples have been extensively studied (Lawal 2017). In the food category, a majority of studies focused on processed products that were expected to be contaminated with PAHs because of heat treatment and/or selected other processing techniques (Mottier et al. 2000; Phillips 1999). However, the number of available reports on PAH contamination in plants is limited. Although PAHs do not show biomagnification potential, they do share certain features with chemicals classified as persistent organic pollutants (POPs). Even if (due to their relatively fast metabolic rate in higher-level organisms) they have never been classified as POPs (Nfon et al. 2008), they are omnipresent in the environment. Similar to other organic pollutants, PAHs are transported through environmental bounds to particulate matter or are dispersed in the gaseous phase (Ziegenhals et al. 2008). Several studies have shown that PAH levels in plants/crops are correlated with the concentration of gaseous and particulate PAHs in the air (Lin et al. 2007; Tao et al. 2006). It might be concluded that atmospheric air is the main route for contamination of plants with PAHs via precipitation and/or exposure to particulate matter (Paris et al. 2018). PAH migration from air to plants depends on several factors, such as the presence of a waxy cuticle and the presence of compounds able to create PAH complexes (Roszko et al. 2018; Liu et al. 2017; Kipopoulou et al. 1999; Lin et al. 2007). Data indicating the other possible route of contamination, i.e., absorption of PAHs via plant roots, are rather limited (Liu et al. 2017; Paris et al. 2018).

PAHs are dangerous chemicals known to produce detrimental health effects in living organisms, including carcino- and genotoxicity in humans and wildlife (Rugen et al. 1989). In Europe, benzo[a]pyrene (BaP) was first proposed as a single marker of total PAH concentration, and its maximum permitted levels were defined for numerous food products. Additionally, 15 other PAHs were indicated, the concentrations of which should be monitored (EC, European Commission 2006). Later, the European Food Safety Authority panel on contaminants in food concluded that BaP and chryzene (Chr), benzo[a]anthracene (BaA), and benzo[b]fluoranthene (BbFl), which are collectively known as PAH 4, were a better indicator of total PAHs than BaP alone. As a consequence, the European Commission (EC) regulations contain maximum limits for both PAH 4 and BaP (EFSA, European Food Safety Authority 2008; FSA, Food Standards Agency 2012).

In the twentieth century, certain organochlorine pesticides (OCPs) such as DDT, HCB, chlordane, dieldrin, endrin, HCH, or heptachlor were used in the majority of the developed countries to protect agricultural crops against insects. These legacy OCPs are classified as POPs (UN, United Nations 2007). In fact, their chemical stability is sufficiently high that their residues are still found in the environment. DDT is still used in certain regions of the world to control mosquitos and thus prevent malaria. In Europe, the maximum DDT levels in various foodstuffs/crops are regulated by EC Regulation 396/2005 (EC, European Commission 2005). OCPs contaminate crops in the same way as other POPs: long-range aerial transport, air precipitation, and binding with particulate matter.

The aims of this study are to (i) determine the background levels of PAHs and legacy OCPs in wheat samples collected in 2017 and 2018 in Poland, (ii) identify differences between the levels for wheat harvested in various regions of Poland, (iii) evaluate differences in contamination sources manifested by profiles of the identified chemicals, (iv) identify possible correlations between different classes of chemicals present in wheat, and (v) assess the health risks associated with the presence of PAHs and OCPs in Polish wheat.

## Materials and methods

### Chemicals/reagents

HPLC-grade solvents were exclusively used in this study. *n*-Hexane, cyclohexane, dichloromethane, ethyl acetate, diethyl ether, toluene, and water were supplied by Sigma-Aldrich (Bellefonte, PA, USA). Analytical-grade anhydrous sodium sulfate was supplied by Avantor (Gliwice, Poland). Silica gel 60 (0.063–200 mm), Florisil 60, was supplied by Merck (Darmstadt, Germany). Bio-Beads SX-3 were purchased from Bio-Rad (Warsaw, Poland). High-purity (> 97%) native PAH standards (naphthalene, acenaphthylene, acenaphthene, fluorene, phenanthrene, anthracene, fluoranthene, pyrene, benzo[c]fluorene, benzo[c]phenanthrene, chrysene, benzo[a]anthracene, cyclopenta[c,d]pyrene, benzo[b]fluoranthene, benzo[j]fluoranthene, benzo[k]fluoranthene, 7,12-dimethylbenz[a]anthracene, benzo[a]pyrene, benzo[e]pyrene, 3-methylcholanthrene, indeno[1,2,3-cd]pyrene, dibenzo[a,h]anthracene, benzo[g,h,i]pyrelene, dibenzo[a,l]pyrene, dibenzo[a,i]pyrene, dibenzo [a,e]pyrene, dibenzo[a,h]pyrene, and 5-methylchryzene) were supplied by Dr. Ehrenstorfer (Augsburg, Germany) and AccuStandard (New Haven, CT, USA). Deuterium-labeled PAH internal standards (naphthalene-d8, pyrelene-d12, chrysene-d12, and phenantren-d10) were supplied by Sigma-Aldrich. Pesticide standards (*o*,*p*-DDT, *p*,*p*-DDT, DDD, DDE, HCB, chlordane, dieldrin, endrin, HCH, and heptachlor) were sourced from IPO (Warsaw, Poland). ^13^C_12_-labeled PCB congeners IUPAC #181 and native PCB #146 were supplied by Cambridge Isotope Laboratories (Andover, MA, USA).

### Test material

The levels of 28 PAHs and 15 OCPs were evaluated in 200 wheat samples (100 harvested in 2017 plus 100 harvested in 2018; each composite sample > 500 g) collected from grain elevators located in 16 districts of Poland. The samples were ground and stored at − 20 °C until the time of analysis.

### Sample preparation

Methods used in this study have been previously detailed (Roszko et al. 2014, 2018). In brief, 20 g of a composite sample was placed in a 250-ml glass Erlenmeyer flask, spiked with labeled internal standards (20 ng each), and extracted twice with 50 ml of acetone:*n*-hexane (1:1 V/V) mixture. Deuterated PAHs were used as internal standards for PAHs, and ^13^C_12_-labeled PCB 181 was used as the internal standard for OCPs. Both extracts were combined, filtered via filter paper into a 100-ml round bottom flask, and evaporated nearly to dryness using a rotary evaporator operated at 40 °C. Residues dissolved in approximately 5 ml cyclohexane-dichloromethane mixture (1:1 V/V) were filtered through a 0.45-μm syringe filter and injected into a gel permeation chromatographic system. Separations were performed on a 500 × 10 glass column filled with Bio-Beads SX-3 styrene-di-vinylo-benzene-based resin (Omnifit, Cambridge, UK). A 2.5-ml sample loop was used. Cyclohexane-dichloromethane (1:1 V/V) was used as the mobile phase and was flowed at 1 ml min^−1^ rate. Fractions eluting between 23 and 60 ml were collected, combined, and evaporated to approximately 2 ml using a rotary evaporator. Half of the remaining solution was transferred to (i) the top of a 10-mm I.D. chromatographic column filled with 5 g of silica gel deactivated with 5% (m/m) of water, and the other half was transferred to (ii) a glass column containing 1.5 of Florisil. The beds were prepared by pouring silica gel and Florisil into columns filled with cyclohexane. Before use, the columns were prewashed with 20 ml of *n*-hexane.

Columns were eluted with (i) 25 ml of *n*-hexane-dichloromethane mixture (1:4) (V/V) or (ii) 1:16 diethyl ether-*n*-hexane (V/V). The solution volume was reduced to approximately 1 ml using a rotary evaporator, spiked with PCB 146 (syringe standard), mixed with toluene to produce a 20% final concentration (extract volume was measured using a glass syringe used in sample retrieval), transferred into a chromatographic vial, and analyzed using a GC/MS/MS system.

### GC/MS analyses

Instrument parameters were identical to those used previously (Roszko et al. 2014, 2018). A Thermo-Finnigan Trace GC Ultra gas chromatograph (Austin, TX, USA) connected via a heated transfer line with a Polaris Q low-resolution ion trap mass spectrometer (Austin, TX, USA) equipped with a programmable temperature vaporizer (PTV)–based injector and TriPlus Autosampler (Austin, TX, USA) were used. Chromatographic separations of PAHs were performed on a 20 × 0.18-mm × 0.18-mm Rtx-17MS fused-silica capillary column (Restek, Bellefonte, PA, USA) connected via a Vu2 Union connector (Restek) to a 5-m × 0.53-mm guard column/retention gap (Restek). In all cases, helium was used as carrier gas at a constant flow of 1.2 ml min^−1^. Samples were introduced via PTV injector operated in solvent split mode. An amount of 35 μl of extract was introduced into a cold injector (30 °C 1 min hold, vent flow of 150 ml min^−1^), and the temperature was subsequently ramped at 14.5 °C min^−1^ to 300 °C (2 min hold). The injector was cleaned at 320 °C for an additional 5 min with vent flow of 100 ml min^−1^. The following temperature program was applied for separation: initial 30 °C (2 min hold), ramped to 180 °C at 15 °C min^−1^, ramped to 190 °C at 2 °C min^−1^, ramped to 290 °C at 2.5 °C min^−1^, and ramped to 320 °C at 5 °C min^−1^ (15 min hold). The mass spectrometer transfer line and ion source were held at 320 °C and 300 °C, respectively. The mass was calibrated against perfluorotributylamine (FC-43) in electron-impact positive ionization mode, in line with the manufacturer’s recommendations. The multiplier bias was 1475 V, and the automatic gain control was set to 50. The mass spectrometer was operated in MS/MS mode with a low excitation voltage setup, as reported previously (Roszko et al. 2018). Statistical parameters of the method are shown in Table 1. LOD and LOQ values were calculated from the chromatograms of the spiked samples (at 0.1 μg kg^−1^). The LOD and LOQ were expressed as theoretical concentrations producing chromatographic peaks with S/N values of 3 and 10, respectively.

**Table 1:** Method statistical parameters

Compound		Nonspiked concentration (μg kg^−1^) (*n* = 3)	Recovery rate *R* (*n* = 9)			Average		LOD	LOQ
			At spiking level (μg kg^−1^)						
			0.1 (%)	1 (%)	5 (%)	*R* (%)	RSD (%)	μg kg^−1^	
PAH	Naphthalene	3.19	N/D	83	95	89	8	0.016	0.055
	Acenaphthylene	0.51		72	84	78	8	0.012	0.04
	Acenaphthene	0.69		83	92	88	6	0.012	0.04
	Fluorene	0.92		83	89	86	4	0.0105	0.035
	Phenanthrene	2.73		94	82	88	8	0.012	0.04
	Anthracene	0.40		83	90	87	5	0.015	0.05
	Fluoranthene	0.33		83	79	81	3	0.006	0.02
	Pyrene	0.42		89	80	85	6	0.0075	0.025
	Benzo[c]fluorene	0.04	87	73	79	76	7	0.0135	0.045
	Benzo[c]phenanthrene	-	78	85	81	83	4	0.0165	0.055
	Chryzene	-	103	89	96	93	7	0.0105	0.035
	Cyclopenta[c,d]pyrene	0.07	85	108	81	95	15	0.0075	0.025
	Benzo[a]anthracene	0.21	89	100	99	100	6	0.021	0.07
	5-Metylchryzene	-	81	73	91	82	9	0.018	0.06
	Benzo[b]fluoranthene	-	85	104	95	99	9	0.018	0.06
	Benzo[j]fluoranthene	0.08	88	108	96	102	10	0.0165	0.055
	Benzo[k]fluoranthene	0.10	73	99	91	95	13	0.0195	0.065
	7,12-Dimethyl[a]anthracene	0.11	72	78	76	77	3	0.009	0.03
	Benzo[e]pyrene	0.91	N/D	112	96	104	11	0.012	0.04
	Benzo[a]pyrene	0.11	90	114	105	110	12	0.0255	0.085
	3-Methylcholanthrene	-	87	105	107	106	11	0.003	0.01
	Indeno[1,2,3-cd]pyrene	0.11	91	105	97	101	7	0.024	0.08
	Dibenzo[a,h]anthracene	-	75	95	97	96	12	0.0255	0.085
	Benzo[g,h,i]pyrelene	-	85	109	90	99	13	0.048	0.16
	Dibenzo[a,l]pyrene	0.03	85	81	99	90	9	0.012	0.04
	Dibenzo[a,i]pyrene	-	79	77	87	82	5	0.012	0.04
	Dibenzo[a,e]pyrene	0.07	78	93	90	92	8	0.0195	0.065
	Dibenzo[a,h]pyrene	0.05	74	90	87	89	9	0.012	0.04
OCP	HCB*	-	79	83	81	82	2	0.02	0.05
	HCH alpha*	-	83	78	88	83	5	0.02	0.05
	HCH beta*	-	81	85	89	87	4	0.02	0.05
	HCH gamma*	-	98	91	89	90	5	0.02	0.05
	HCH delta*	-	86	80	89	85	5	0.02	0.05
	Heptachlor*	-	75	78	82	80	4	0.02	0.05
	Heptachlor epoxide*	-	79	83	81	82	2	0.02	0.05
	Chlordan cis*	-	90	88	86	87	2	0.02	0.05
	Chlordan trans*	-	85	83	86	85	2	0.02	0.05
	Dieldrin*	-	79	83	81	82	2	0.02	0.05
	*p*,*p*-DDE	0.29	78	83	85	84	4	0.03	0.010
	*o*,*p*-DDT	0.14	75	88	89	89	8	0.02	0.08
	*p*,*p*-DDT	0.06	83	88	85	87	3	0.02	0.05
	*p*,*p*-DDD	0.07	88	93	90	92	3	0.02	0.05
	Endrin*	-	82	80	85	83	3	0.02	0.05

*N*/*D* not determined due to high difference between spike and blank concentration

*LOQ and LOD values taken from the lowest points of the respective calibration curves

### Data analysis

The Xcalibur 1.2 software was used to acquire and analyze data. The reported concentrations are the mean values of two parallel determinations (± 1 SD, if given). Concentrations below LOQ were accounted for in two ways: assumed as zero (such results are hereafter referred to as “lower bound”) or assumed as LOQ/2 (“medium bound”). The results were statistically assessed in terms of analysis of variance and principal component analysis (Statistica 9.0 software suite).

## Results and discussion

### PAH levels and profiles

**Fig. 1:**
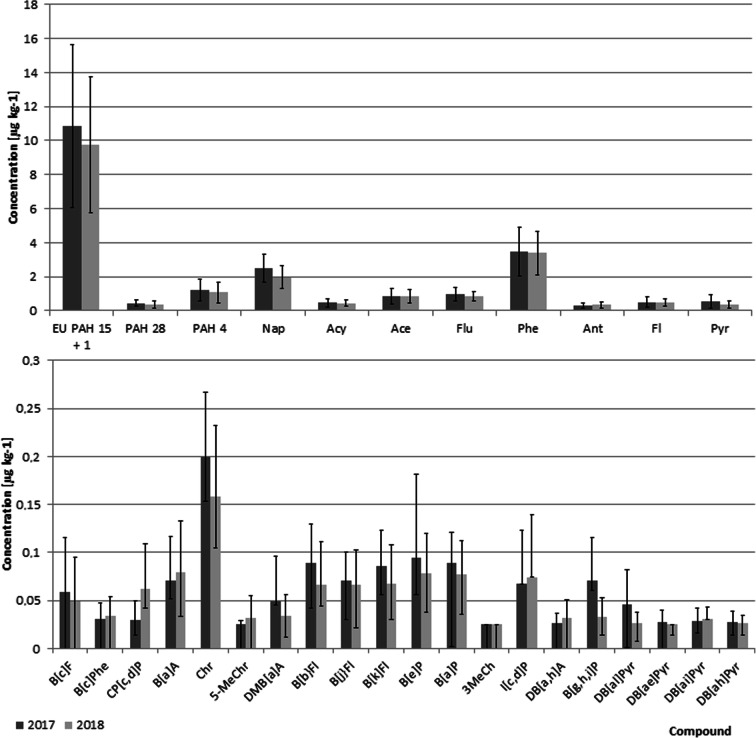
Comparison of PAH profiles in grains harvested in 2017 and 2018

The mean/median/max concentrations of individual PAHs calculated from data collected on all 200 tested samples are shown in Table 2. Comparison between 2017 and 2018 vegetation seasons and average PAH profiles are shown in Fig. 1. Generally, the observed PAH levels were low.

**Table 2:** Mean/median/max concentrations of individual PAHs calculated from data collected on all 200 tested samples (see the “Data analysis” section for explanation of “lower/medium bound”)

Compound	Lower bound (μg kg^−1^)				Medium bound (μg kg^−1^)				Positive samples (%)
	Mean	Median	Max	SD	Mean	Median	Max	SD	
Nap	2.24	2.16	4.84	0.78	2.24	2.16	4.84	0.78	100
Acy	0.46	0.42	1.61	0.22	0.46	0.42	1.61	0.22	100
Ace	0.87	0.79	2.16	0.41	0.87	0.79	2.16	0.41	100
Flu	0.90	0.84	2.28	0.35	0.90	0.84	2.28	0.35	100
Phe	3.47	3.25	8.66	1.34	3.47	3.25	8.66	1.34	100
Ant	0.32	0.28	0.87	0.15	0.32	0.28	0.87	0.15	100
Fl	0.51	0.45	2.29	0.26	0.51	0.45	2.29	0.26	100
Pyr	0.45	0.38	2.18	0.30	0.45	0.38	2.18	0.30	100
B[c]F	0.06	0.05	0.29	0.05	0.05	0.03	0.29	0.05	43
B[c]Phe	0.04	0.03	0.11	0.02	0.03	0.03	0.11	0.02	16
CP[c,d]P	0.04	0.03	0.21	0.05	0.05	0.03	0.21	0.04	29
B[a]A	0.08	0.07	0.24	0.05	0.08	0.07	0.24	0.05	67
Chr	0.18	0.18	0.46	0.07	0.18	0.18	0.46	0.07	99
5-MeChr	0.02	0.01	0.18	0.02	0.03	0.03	0.18	0.02	7
DMB[a]A	0.02	0.00	0.24	0.05	0.04	0.03	0.24	0.04	21
B[b}Fl	0.08	0.07	0.21	0.04	0.08	0.07	0.21	0.04	75
B[j}Fl	0.07	0.07	0.17	0.03	0.07	0.07	0.17	0.03	76
B[k]Fl	0.08	0.07	0.21	0.04	0.08	0.07	0.21	0.04	79
B[e]P	0.09	0.08	0.91	0.07	0.09	0.08	0.91	0.07	85
B[a]P	0.09	0.08	0.20	0.03	0.08	0.08	0.20	0.03	90
3-MeCh	0.00	0.00	0.02	0.01	0.03	0.03	0.03	0.00	0
I[c,d]P	0.07	0.06	0.45	0.06	0.07	0.06	0.45	0.06	59
DB[a,h]A	0.01	0.00	0.14	0.02	0.03	0.03	0.14	0.02	6
B[g,h,i]P	0.05	0.05	0.24	0.04	0.05	0.03	0.24	0.04	47
DB[al]Pyr	0.03	0.01	0.20	0.03	0.04	0.03	0.20	0.03	19
DB[ae]Pyr	0.00	0.00	0.10	0.01	0.03	0.03	0.10	0.01	2
DB[ai]Pyr	0.01	0.00	0.14	0.02	0.03	0.03	0.14	0.01	12
DB[ah]Pyr	0.01	0.00	0.09	0.02	0.03	0.03	0.09	0.01	5
PAH 28	10.26	9.74	23.68	3.25	10.38	9.88	23.87	3.27	N/A
PAH 4	0.43	0.40	1,01	0.16	0.42	0.40	1.01	0.17	
PAH 27	8.02	7.58	21.38	2.84	8.14	7.71	21.57	2.86	
EU PAH 15 + 1	1.03	0.99	2.43	0.38	1.15	1.11	2.46	0.38

Mostly low molecular weight hydrocarbons were found with a predominance of naphthalene, phenanthrene, fluorene, acenaphthene, acenaphthylene, fluoranthene, pyrene, and anthracene. For approximately half of the remaining higher molecular weight PAHs, the medium-bound results are higher than the lower-bound results, which reflects contributions from concentrations lower than the LOQ.

The maximum permissible PAH levels in wheat grains were not specified in EC Regulation 1881/2006 (EC, European Commission 2006) or in EC Regulation 835/2011, which amended the former version (EC, European Commission 2011). The 1-μg kg^−1^ lowest level specified in those Regulations addressed BaP and PAH 4 in processed cereal-based foods and baby foods. In the tested wheat grains, the mean concentrations of both BaP and PAH 4 were lower than the given threshold: 0.09 μg kg^−1^ for the former and 0.43 μg kg^−1^ for the latter. The highest observed PAH 4 concentration slightly exceeded the threshold in only a single sample (1.01 μg kg^−1^).

At the beginning of any wheat processing, the grains are commonly separated from their external most heavily contaminated components (e.g., bran) (Roszko et al. 2014), which might significantly reduce food contamination.

The PAH profiles determined in grains harvested in 2017 and 2018 were generally similar, as shown in Fig. 1. No statistically significant differences (*α* = 0.05) were observed between the mean concentrations of either individual PAHs or PAH markers. However, the scatter of the concentrations was rather high.

The above results are generally in line with reports published in the literature (although literature data on PAHs in unprocessed wheat grains are scarce). Kobayashia et al. (2008) observed 2- to 4-ring PAHs in wheat grain samples harvested in various US locations. Similar to the results of this study, naphthalene and phenanthrene were the most abundant compounds. A rather high scatter was observed in the naphthalene concentrations. The authors suggested that vehicle exhaust gases were the major source of the studied PAH contamination and that the observed differences in naphthalene concentration probably reflected different intensities of emission of this chemical in regions with and without nearby cities that generate heavy traffic. Similar conclusions were drawn by Liu et al. (2017), who studied wheat samples harvested in China and found mostly low molecular PAHs at rather low concentrations.

**Fig. 2:**
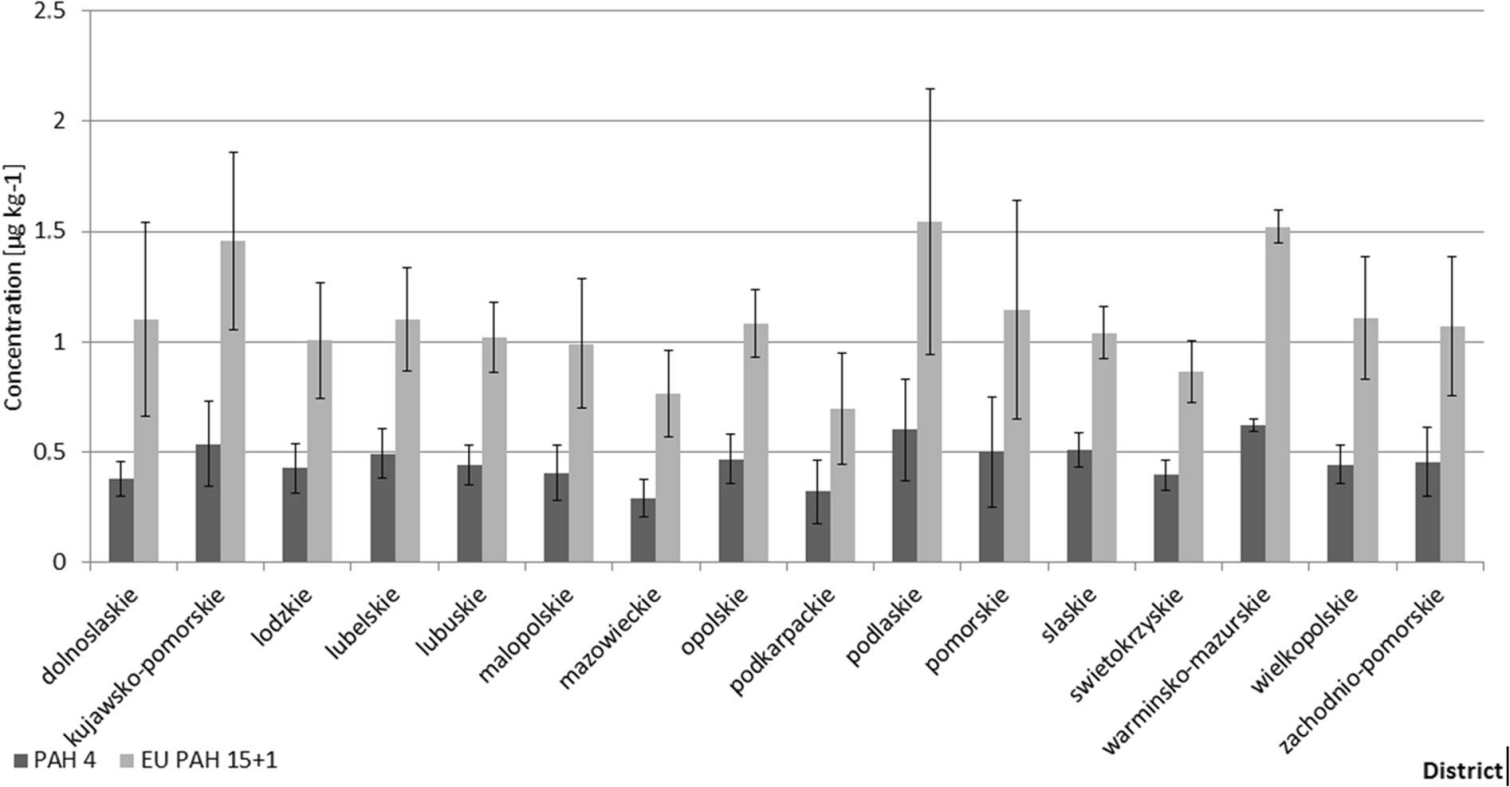
Breakdown of PAH 4 and EU PAH 15 + 1 mean concentrations in 2017 samples by district in Poland

**Fig. 3:**
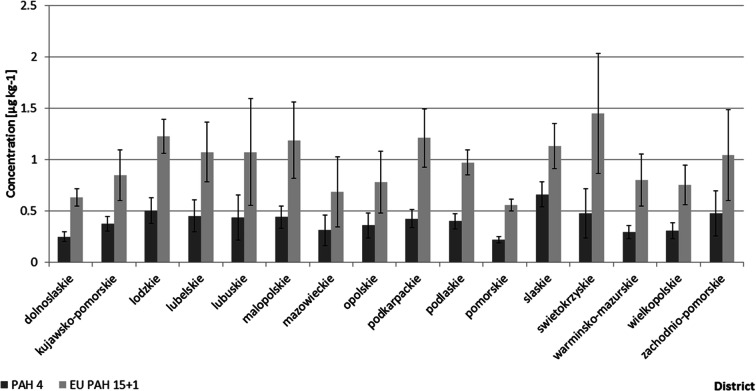
Breakdown of PAH 4 and EU PAH 15 + 1 mean concentrations in 2018 samples by district in Poland

**Fig. 4:**
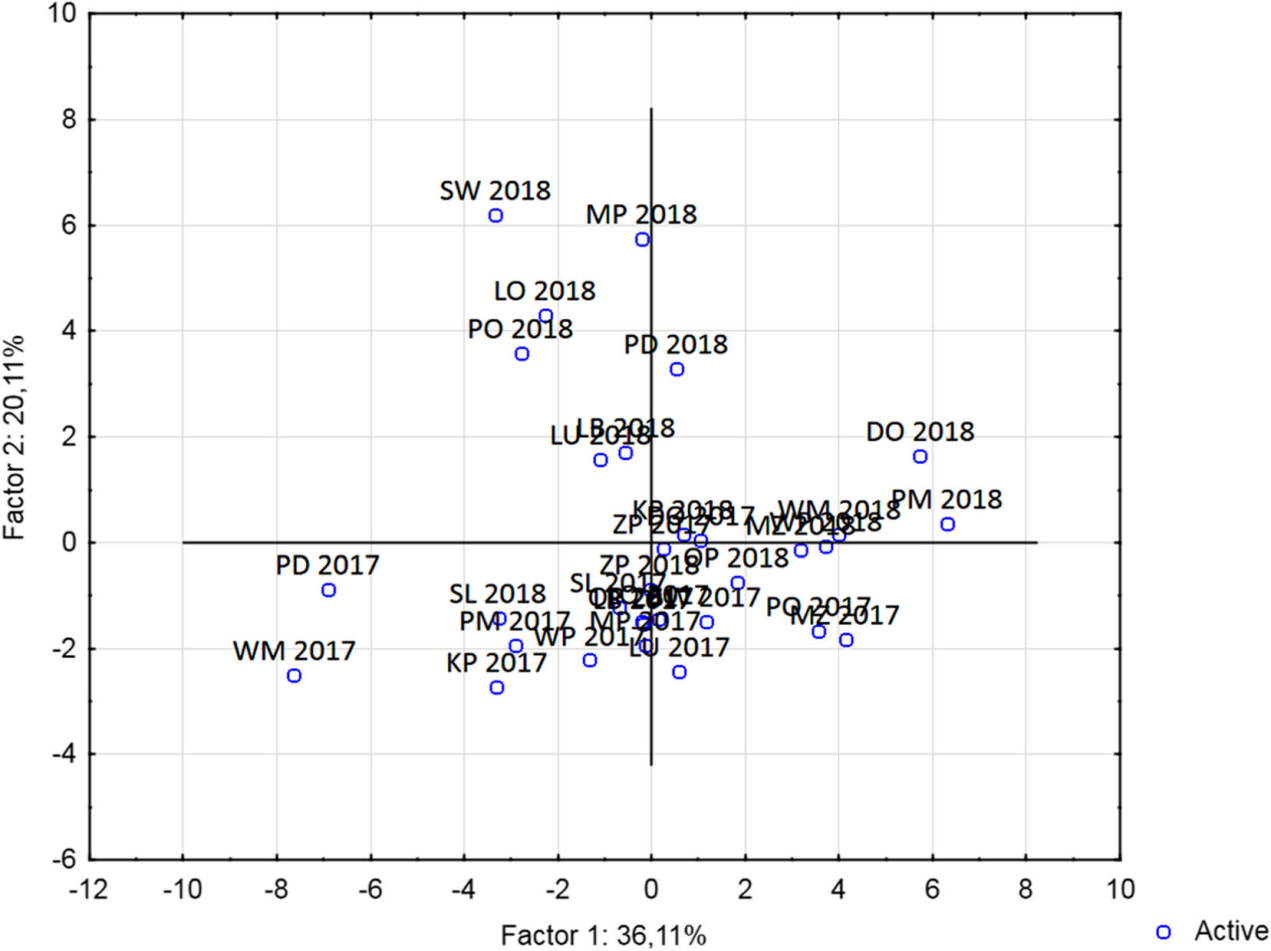
Principal component analysis scatter plot of PAH profiles in wheat harvested in individual districts of Poland. DO dolnoslaskie; KP kujawsko-pomorskie; LD lodzkie; LU lubelskie; LB lubuskie; MP malopolskie; MZ mazowieckie; OP opolskie; PO podkarpackie; PD podlaskie; PM pomorskie; SL slaskie; SW swietokrzyskie; WM warminsko-mazurskie; WP wielkopolskie; ZP zachodnio-pomorskie

Various districts of Poland have significantly different population densities, but we did not identify any statistically significant differences in PAH contamination of wheat samples harvested in different districts. In most cases, samples from the same district showed a high variability of concentrations with low absolute values (see Figs. 2 and 3). Additionally, we did not identify any significant differences between PAH profiles from different districts. Principal component analysis scatter plots for the studied districts are shown in Fig. 4. Selected visible grouping/clustering is not attributable to geographical locations/seasons, which suggest that variability of the contamination sources significantly affects the observed PAH profiles.

**Fig. 5:**
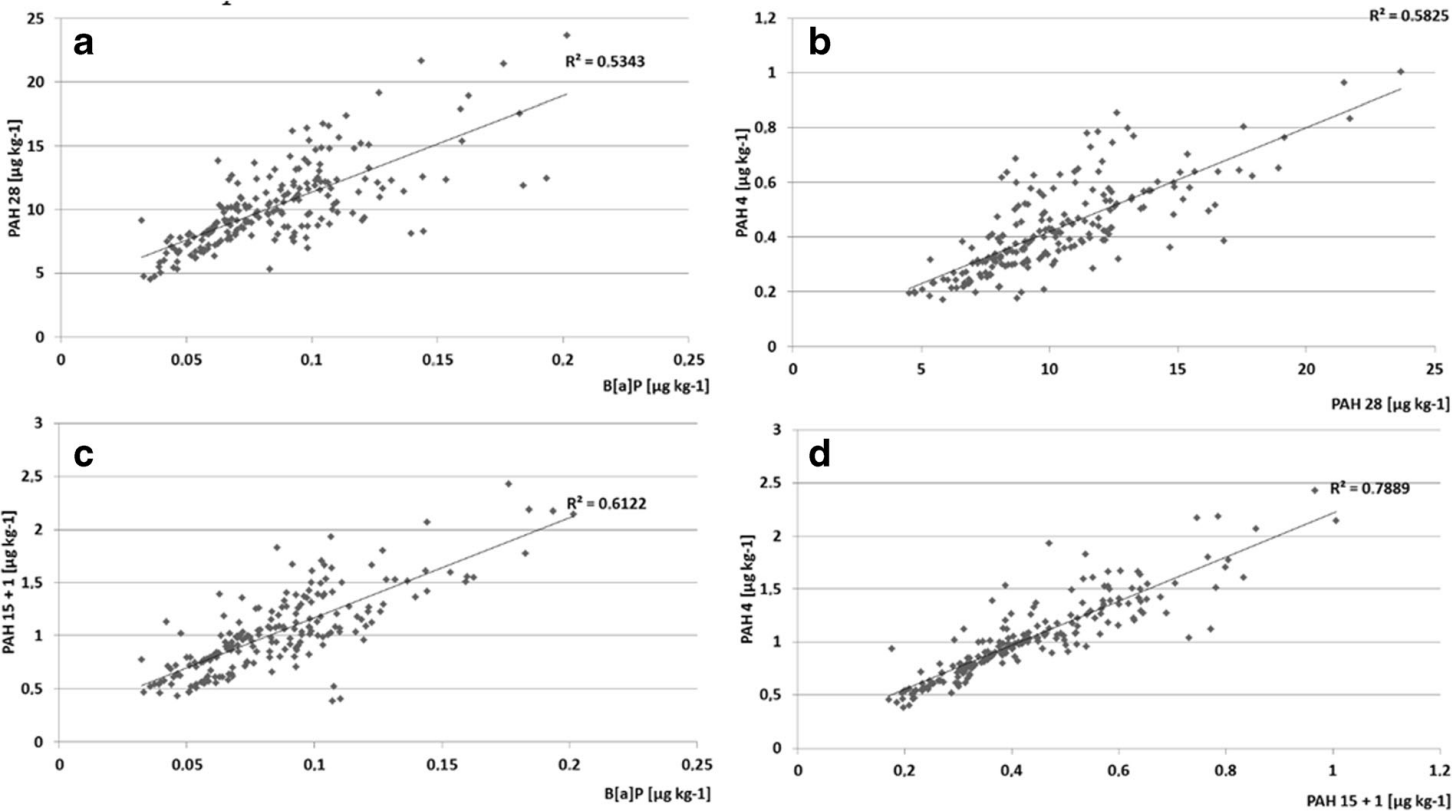
Correlations between PAH 28 and BaP (**a**), PAH 4 and PAH 28 (**b**), EU PAH 15 + 1 and BaP (**c**), and PAH 4 and EU PAH 15 + 1 (**d**)

In numerous cases, PAH concentrations were statistically significantly correlated (*α* = 0.05). The strongest correlations were found between PAH 4 and PAH 15 + 1 (*R*^2^ = 0.78, see Fig. 5d), and between PAH 15 + 1 and BaP (*R*^2^ = 0.61, see Fig. 5c). The former correlation is not unexpected because both groups include the same compounds. PAH 4/PAH 28 and PAH 28/BaP correlations were somewhat weaker but still statistically significant. The former indicates that PAH 4 might be a good indicator of contamination with low molecular PAHs because the PAH 28 group includes many low molecular PAHs. Weaker correlations between groups covering both low and high molecular weight PAHs might be explained by the fact that the former might more easily translocate through air due to higher vapor pressure than the latter.

**Fig. 6:**
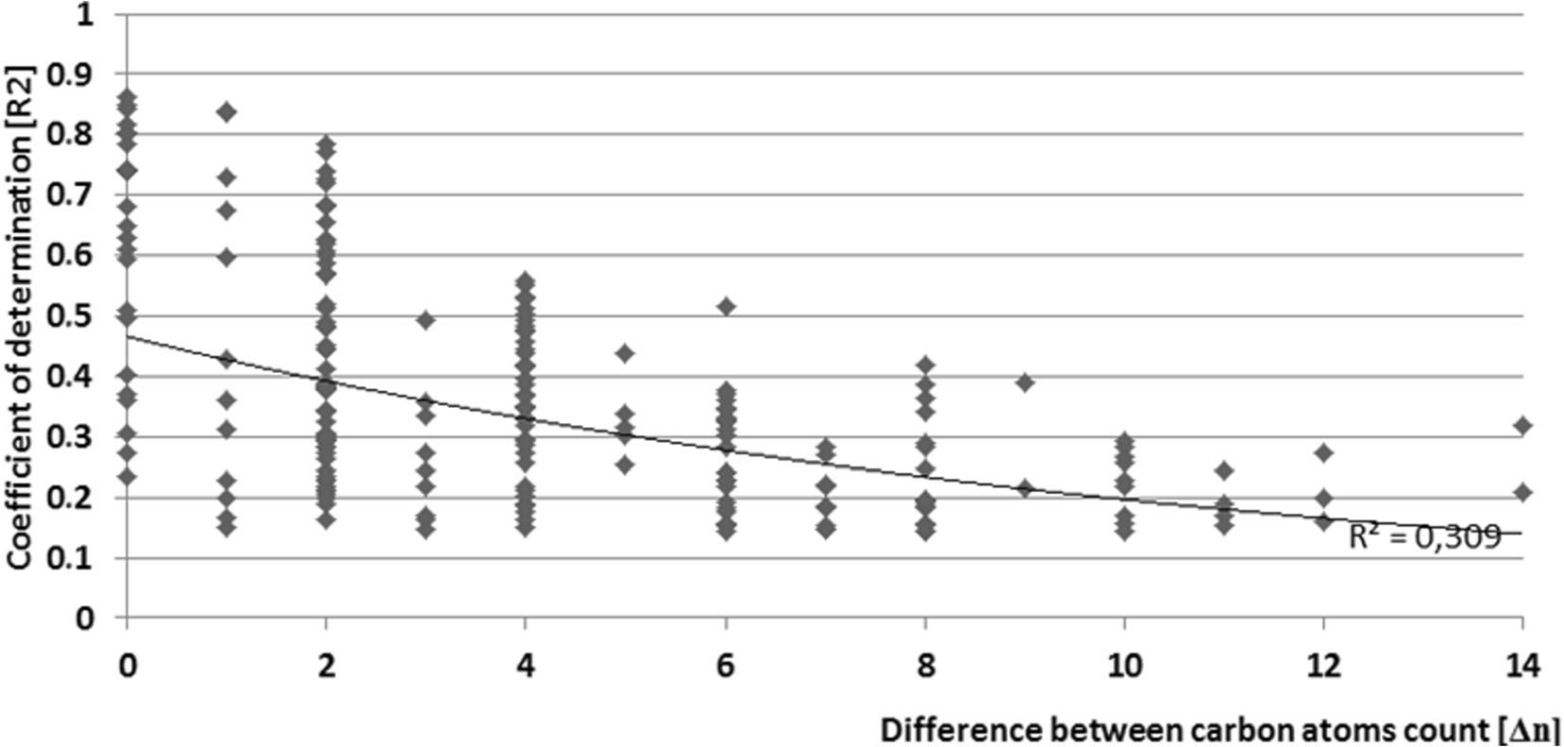
Strength of correlation between different PAHs vs. difference between carbon atom count in rings of both PAHs in question

We observed that the correlations were higher for the more similar molecular weights of PAHs in question (Fig. 6). However, it must be noted that the lack of correlation might also be caused by the different origins of the PAHs, i.e., profiles of petrogenic, biological, and pyrogenic PAHs might differ significantly, and the PAH profiles and correlations between them depend on the relative contributions of these three sources to the contamination (Abdel-Shafy and Mansour 2016).

### Identification of PAH contamination sources based on diagnostic ratios

Various methods/indicators have been proposed to identify environmental sources of the PAHs found in samples (Yunker et al. 2002; Yunker et al. 2011; Saclo et al. 2000; Magi et al. 2002; Chen and Chen 2011; Chen et al. 2013). Yunker et al. (2002) proposed differentiation of the sources based on the ratios of concentrations of selected PAHs. The ratios are influenced by different thermodynamic characteristics (including formation heat) of individual PAHs. In particular, petrogenic (oil extraction) and pyrogenic (combustion of petroleum/wood/biomass/coal) contamination can be differentiated in this manner. Such an approach assumes that various PAHs are transformed and degraded at the same rate during their lifetimes in the environment, such that source-characteristic concentration ratios are preserved (Biache et al. 2014; Clement et al. 2015). However, Santos et al. (2017) noted that various ratios might show variable sensitivity and each could be differently affected by PAH transformations in the environment (photooxidation, photolysis). A number of authors have warned that the ratios should be used with caution because of complexity of emission sources and possible transformations in the environment (Yunker et al. 2002; Daskalou et al. 2009; Tobiszewski and Namiesnik 2012).

The diagnostic ratios commonly used to identify sources of PAH contamination include Phen/Ant, Ant/(Phen + Ant), and Flu/(Flu + Pyr) (Magi et al. 2002; Chen and Chen 2011; Chen et al. 2013; Zhang et al. 2004; Qiao et al. 2006; Chen et al. 2013). Yunker et al. (2011) also proposed the use of a number of other diagnostic ratios, including BaA/BaA + Chr and BF/BF + B[e]P. The four ratios selected in this study are BaA/BaA + Chr, BF/BF + B[e]P, Phen/Phen + Ant, and Flu/Flu + Pyr. The thresholds/ranges of the selected ratios attributed by Yunker et al. (2011) to different PAH contamination sources are shown in Table 3.

**Table 3:** Thresholds/ranges of selected diagnostic ratios attributed by Yunker et al. (2011) to different PAH contamination sources: petroleum, combustion of liquid fossil fuel/mixed sources, and combustion of solid fuel (grass/wood/biomass)/coal)

PAH diagnostic ratio	PAH contamination source		
	Petroleum	Liquid fossil fuel combustion/mixed sources	Grass/wood/biomass/coal combustion
Ant/(Phn + Ant)	< 0.10	> 0.10	> 0.10
Flu/(Flu + Pyr)	< 0.40	0.40–0.50	> 0.50
BaA/(BaA + Chr)	< 0.20	0.20–0.35	> 0.35
BF/(BF + BeP)	< 0.50	0.50–0.70	> 0.70

**Fig. 7:**
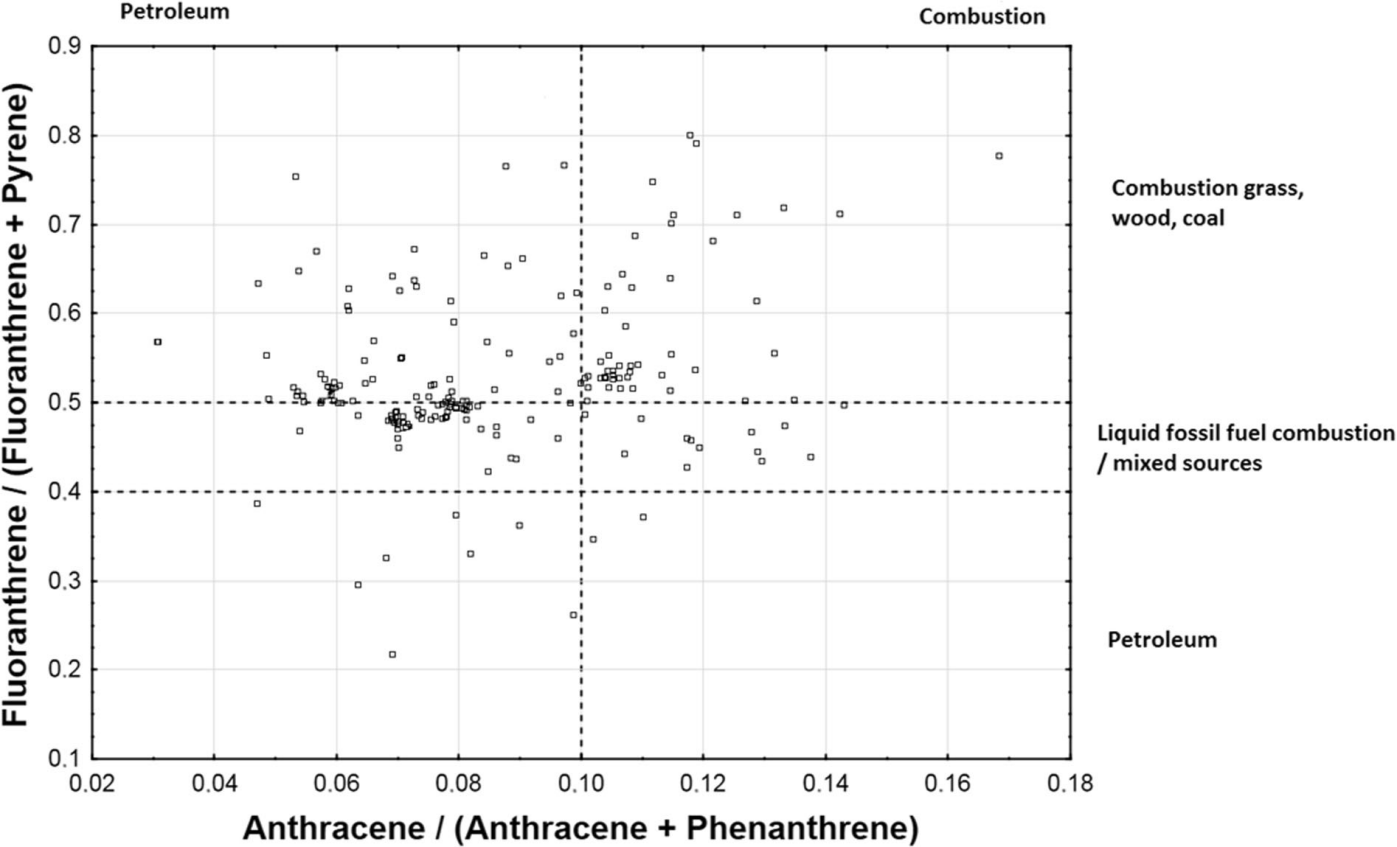
Scatterplot of Flu/(Flu + Pyr) and Ant/(Ant + Phen) PAH diagnostic ratios with marked regions corresponding to different emission sources

**Fig. 8:**
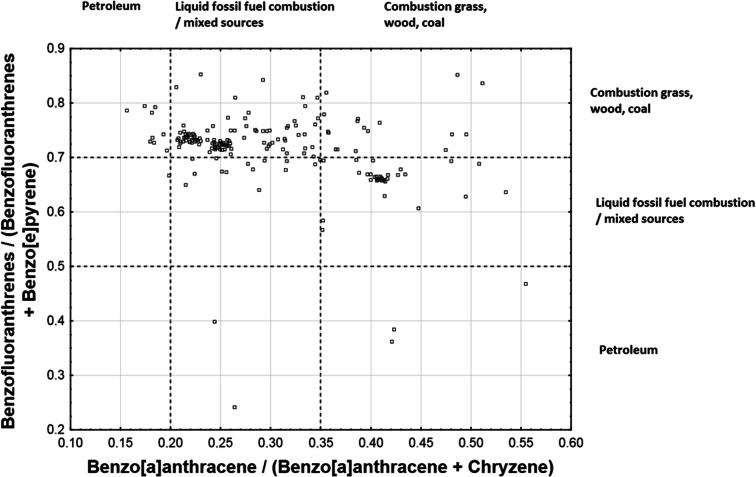
Scatterplot of BF/(BF + BeP) and BaA/(BaA + Chr) PAH diagnostic ratios with marked regions corresponding to different emission sources

Scatterplots of the four PAH diagnostic ratios selected in this work are shown in Figs. 7 and 8. The Flu/(Flu + Pyr) ratio fell below 0.4 in only a few percent of our samples, ranged from 0.4 to 0.5 in a significant portion of the samples, and exceeded 0.5 in a majority of the samples. This result indicates combustion as the main source of contamination. However, the Ant/(Ant + Phen) ratio did not exceed 0.1 in a large percentage of the samples, which indicates petrogenic origin of the contamination in those samples. The BaA/(BaA + Chr) ratios fell below 0.2 in only a few percent of the samples and within the 0.2–0.35 range in a majority of the samples. This result also indicates combustion as the main source of contamination. The BF/(BF + BeP) ratio was greater than 0.7 in a majority of the samples, indicating more precisely the combustion of grass/wood/biomass/coal as the contamination source rather than combustion of liquid fossil fuels/mixed sources.

Generally, our results indicate that in a majority of cases, PAH contamination in Polish wheat grains can be traced to combustion of coal, liquid fossil fuels, and/or biomass (wood, grass etc.).

### Organochlorine pesticides

**Fig. 9:**
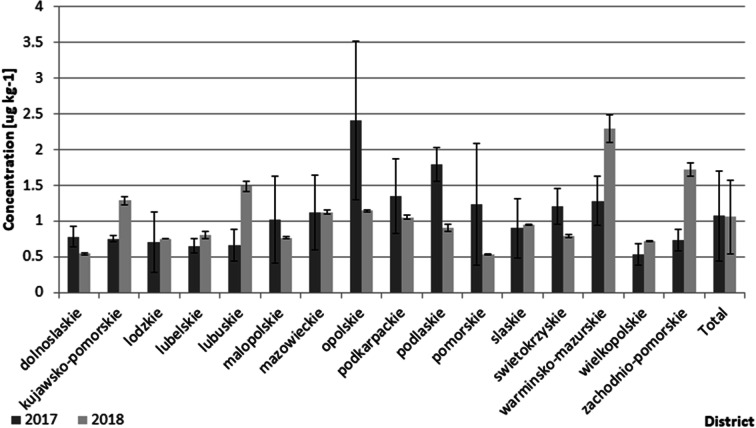
Breakdown of OCP mean concentrations in 2017 and 2018 samples by district in Poland. The two last data points are for the entire country of Poland

In the group of legacy pesticides studied in this work, only DDT isomers and their degradation products (DDE and DDD) were found at concentrations exceeding the LOQ of the applied analytical method. The mean/median/min/max concentrations of individual pesticides calculated from data collected on all 200 tested samples are shown in Table 4. The breakdown of OCP concentrations in the 2017 and 2018 samples by district in Poland is shown in Fig. 9.

**Table 4:** Mean/median/min/max concentrations of OCPs in the tested wheat samples

Compound	Concentration (μg kg^−1^)									
	Lower bound					Medium bound				
	Mean	Median	Min	Max	SD	Mean	Median	Min	Max	SD
*p*,*p*-DDE	0.37	0.32	0.11	1.42	0.20	0.37	0.32	0.11	1.42	0.20
*o*,*p*-DDT	0.32	0.26	0.00	1.51	0.24	0.32	0.26	0.03	1.51	0.24
*p*,*p*-DDT	0.22	0.13	0.00	2.03	0.26	0.22	0.13	0.01	2.03	0.26
*p*,*p*-DDD	0.17	0.14	0.00	1.23	0.15	0.17	0.13	0.03	1.23	0.15
Total DDT/E/D	1.07	0.85	0.31	5.06	0.68	1.08	0.85	0.343	5.06	0.68

Generally, the OCP concentrations in our samples were low (similar to the PAH concentrations). The mean lower-bound concentration of total DDT + metabolites was 1.07 ± 0.68 μg kg^−1^ (range 0.31–5.06 μg kg^−1^). These values are far below the 50 μg kg^−1^ MRL value set in Europe Regulation 396/2005 (EC, European Commission 2005) and are lower that the data from our previous surveillance studies focused on cereal-based products (Roszko et al. 2014). In the latter paper, the mean concentrations of individual isomers were in the microgram per kilogram range. However, it must stressed that cereal-based products and not wheat grains were studied in that work. Toteja et al. (2006) reported much higher DDT concentrations in wheat harvested in India with a median of 13 μg kg^−1^ and a maximum of 7000 μg kg^−1^ in addition to the detectable quantities of HCH that were found. Łozowicka and Kaczyński (2009) reported generally low levels of DDT and its metabolites in agricultural crops harvested in Poland. Only a single sample out of the 275 samples tested was considered positive and contained more than 5 μg kg^−1^ of DDTs.

**Fig. 10:**
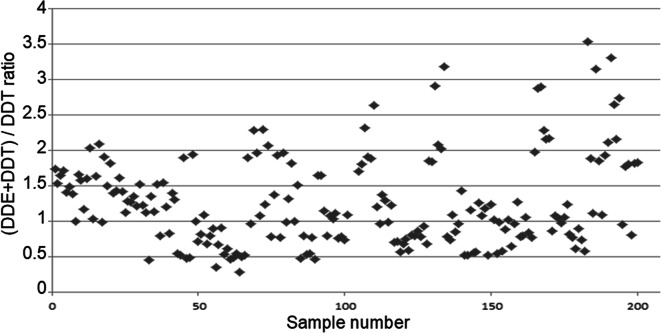
Scatterplot of (DDE + DDD)/DDT ratios calculated for all 200 tested samples

**Fig. 11:**
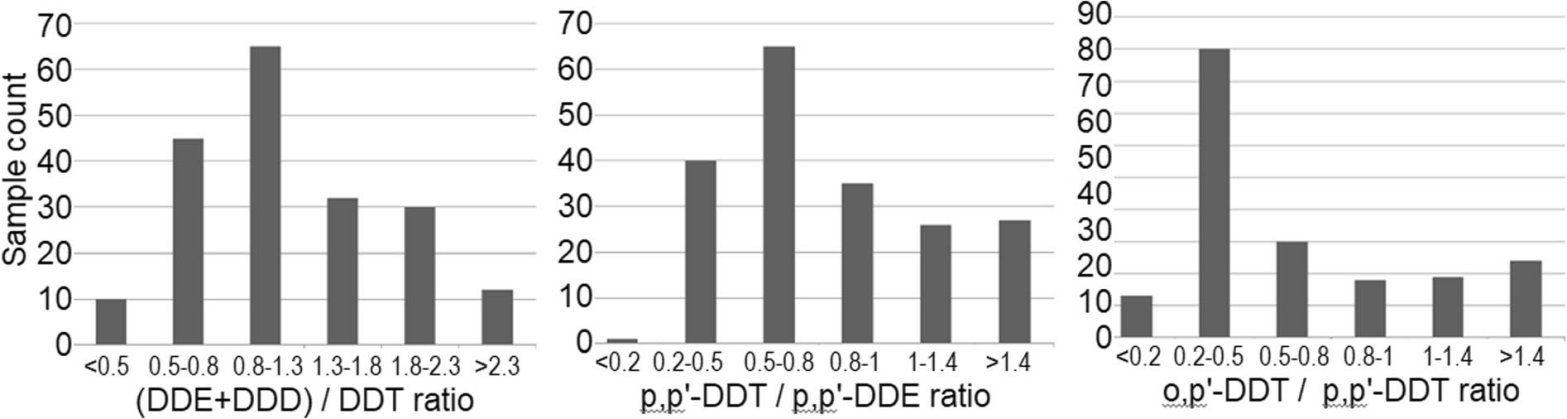
Frequency histograms of selected OCP ratios

The main DDT degradation products include DDE and DDD (Wang et al. 2008; Okay et al. 2011; Zhang et al. 2018). The composition of these compounds and their concentration ratios have been reported as possible indicators of pollution sources (Tao et al. 2006). However, Zhang et al. (2018) reported that different behaviors of individual compounds in specific matrices (such as various types of soil) might lead to fractionation of certain compounds, which might modify the native DDT ratios. As a rule of thumb, a (DDE + DDD)/ΣDDT ratio above 0.5 is taken as a sign of long-term degradation/fractionation of DDT (Hites and Day 1992). However, Yohannes et al. (2013) and Qiu et al. (2005) noted that the rule is limited to regions in which a given dicofol pesticide containing DDT among the impurities was used at a given time in the past. The (DDE + DDD)/ΣDDT ratios found in this study were within the 0.28–3.5 range, and 95% of the ratios were above 0.5 (see Figs. 10 and 11). Such values suggest residues of older environmental pollution rather than new sources.

Liu et al. (2009) and Zhang et al. (2009) suggested that the *p,p*′-DDT/*p,p*′-DDE ratio could also be used to differentiate new from old sources of OCP pollution. In our samples, the ratio ranged from 0.15 to 3.11, and in a majority of cases, it remained below 1. Even if this result suggests pollution from old environmental residues, the number of cases classified as polluted from new sources was higher than those classified so based on the (DDE + DDD)/DDT ratio (Fig. 11). According to Qiu et al. (2005), an increased *o*,*p*′-DDT/*p*,*p*′-DDT ratio in environmental samples indicates pollution originating in dicofol-containing pesticides. Harner et al. (2004) noted that the ratio of both DDTs spanned a range from 1:5 in selected technical mixtures to 7:1 in dicofol. In our samples, the ratio ranged from 0.044 to 4.1. In majority of cases, the ratio remained below 0, indicating contamination related to certain technical mixtures, and contamination related to dicofol was much less frequent. Becker et al. (2012) studied OCPs in the arctic atmosphere and observed a decline in the *p*,*p*′-DDT and *o*,*p*′-DDT/*p*,*p*′-DDT ratio, reflecting a shift from DDT technical mixtures to dicofol. Those researchers commented that the ratios they observed might reflect ongoing re-emission of the compounds from the soil, climate change, and other factors. The observed environmental background of these chemicals is most likely related to ongoing use of dicofol in Asia, South America, and Southern Europe, as well as to ongoing use of selected DDT technical mixtures (Becker et al. 2012).

### Dietary risk assessment

Analytical data obtained in this study were used to roughly estimate the health risks associated with consumption of PAH/DDT-contaminated wheat grains and/or cereals. Approximately 5.3 million metric tons of grains are consumed each year in Poland (KOWR 2013) in the form of bakery products, pasta, groats, beer, etc. Processing clearly influences the contamination levels in the final products because the outer portions of the grains are commonly removed (see data reported by EFSA for processed and unprocessed cereals, EFSA, European Food Safety Authority 2008). However, heat treatment applied during food processing (e.g., the bread baking process) might produce elevated levels of PAHs (Ciecierska and Obiedziński 2013). Nevertheless, to assess the share of background contamination of grains in dietary risk, the following assumptions were adopted: (i) contamination levels in processed cereals reflect the levels in unprocessed grain, (ii) the average consumption rate is 138 kg person^−1^ year^−1^ (5.3 million tons divided by 38.4 million inhabitants of Poland), (iii) the PAH/DDT contamination level in wheat (approximately 65% of the market) reflects contamination of other grains (rye, oat, etc.), and (iv) the average human body weight is 60 kg.

In the worst-case scenario, 28PAH/PAH 4/B[a]P/ΣDDT intake could be 0.15/0.006/0.001/0.03 μg kg^−1^ b.w. day^−1^, respectively (as calculated from 23/1.01/0.2/5.06 μg kg^−1^, respectively, the maximum concentrations found in this work). For comparison, EFSA reported the following dietary exposure rates of the general population in European countries (average/high values, respectively): 0.00391/0.00648 μg kg^−1^ b.w. day^−1^ for BaP and 0.0195/0.0345 μg kg^−1^ b.w. day^−1^ for PAH 4 (EFSA 2008). In view of the high consumption rate of cereals, their input to total dietary exposure (mean for European Union member states) is high: approximately 24% for BaP and 16% for PAH 4 (EFSA, European Food Safety Authority 2008). For comparison, our results indicate that the share of cereals in the exposure is 32–19% for BaP and 18–32% for PAH 4, depending on the adopted scenario and assuming a fixed total intake value of PAHs. Calculations based on the mean concentrations rather than the maximum concentrations give an 8–14% share of cereals in total intake of BaP and a 9–15% share of cereals in total intake of PAH 4. Even if the concentration data used in the calculations are overestimated (because processing does decrease PAH contamination) and the worst-case scenario is improbable, cereals significantly contribute to the total dietary exposure to PAHs.

Even if the Scientific Committee on Food concluded in 2002 that several PAHs were potentially genotoxic and carcinogenic to humans, no tolerable daily intake thresholds for these compounds have been set so far (EFSA, European Food Safety Authority 2008; FSA, Food Standards Agency 2012). However, to better assess the risk resulting from dietary exposure to PAHs, the European Food Safety Authority’s Panel on Contaminants in the Food Chain adopted the margin of exposure (MOE) approach with respect to BaP and PAH 4. Reference data used in risk calculation were based on reports by Culp et al. (1998) covering the compounds for which oral carcinogenicity data were available (EFSA, European Food Safety Authority 2008). BMDL10 at 0.07 and 0.34 mg kg^−1^ b.w. day^−1^ for BaP and PAH 4 (respectively) were adopted. EFSA concluded that MOE values that might be a health concern were close to or less than 10,000. MOE values calculated for cereals using only dietary risk worst-case estimates reported in this study exceeded 50,000 for both BaP and PAH 4. Even assuming the lowest share in the total intake (18% and 19%), the MOE values would still exceed 10,000. As shown, the health risks resulting from exposure to PAHs taken in with grains might be considered as low (with due regard to the assumptions and limitations of this estimate).

The tolerable daily intake for DDT and its metabolites has been estimated at 10 μg kg^−1^ b.w. day^−1^ (Vromman et al. 2014). The 0.03 μg kg^−1^ b.w. day^−1^ daily intake calculated from the maximum DDT levels found in our samples (the worst-case scenario) is only 0.3% of the TDI value. Exposures below 15% of TDI are viewed as the third priority (of low concern). Health risks resulting from exposure to DDT and its metabolites taken in with Polish wheat grains might be considered as insignificant.

## Conclusions

The concentrations of PAHs and OCPs in wheat produced in Poland are relatively low. All 200 tested samples were compliant with the currently standing European food regulations. The maximum PAH level set for cereal-based baby food was exceeded only in one sample (0.5%). The PAH profiles suggest contamination from combustion-related emission sources (liquid fossil fuels, coal, biomass). Contamination with DDT and its metabolites reflects historical use of technical mixtures containing these compounds rather than any new pollution sources. Neither the contamination rate nor the profiles were diversified throughout Poland. Assessment of dietary risk has shown that the presence of the two contaminant classes in Polish wheat grains is of low concern.

## References

[CR1] Abdel-Shafy HI, Mansour MSM (2016). A review on polycyclic aromatic hydrocarbons: source, environmental impact, effect on human health and remediation. Egyptian Journal of Petroleum.

[CR2] Becker S, Halsall CJ, Tych W, Kallenborn R, Schlabach M, Manø S (2012). Changing sources and environmental factors reduce the rates of decline of organochlorine pesticides in the Arctic atmosphere. Atmospheric Chemistry and Physics.

[CR3] Biache AC, Mansuy-Huaulta L, Faurea P (2014). Impact of oxidation and biodegradation on the most commonly used polycyclic aromatic hydrocarbon (PAH) diagnostic ratios: implications for the source identifications. Journal of Hazardous Materials.

[CR4] Bryła M, Ksieniewicz-Wozniak E, Yoshimari T, Waskiewicz A, Szymczyk K (2019). Contamination of wheat cultivated in various regions of Poland during 2017 and 2018 agricultural seasons with selected trichothecenes and their modified forms. Toxins.

[CR5] Chen CW, Chen CF (2011). Distribution, origin, and potential toxicological significance of polycyclic aromatic hydrocarbons (PAHs) in sediments of Kaohsiung Harbor, Taiwan. Marine Pollution Bulletin.

[CR6] Chen C, Chen C, Dong C, Kao C (2013). Assessment of toxicity of polycyclic aromatic hydrocarbons in sediments of Kaohsiung Harbor, Taiwan. Science of the Total Environment.

[CR7] Ciecierska M, Obiedziński MW (2013). Polycyclic aromatic hydrocarbons in the bakery chain. Food Chemistry.

[CR8] Clement N, Muresan B, Hedde M, Francois D (2015). PAH dynamics in roadside environments: influence on the consistency of diagnostic ratio values and ecosystem contamination assessments. Science of the Total Environment.

[CR9] Culp SJ, Gaylor DW, Sheldon WG, Goldstein LS, Beland FA (1998). A comparison of the tumours induced by coal tar and benzo[*a*]pyrene in a 2-year bioassay. Carcinogenesis.

[CR10] Daskalou V, Vreca P, Muri G, Stalikas C (2009). Recent environmental changes in the shallow Lake Pamvotis (NW Greece): evidence from sedimentary organic matter, hydrocarbons, and stable isotopes. Achieves of Environmental Contamination and Toxicology.

[CR11] EC, European Commission, (2005). Regulation No 396/2005 of the European Parliament and the Council of 23 February 2005 on maximum residue levels of pesticides in or on food and feed of plant and animal origin and amending. Council Directive 91/414/EEC, *Official Journal of the European Union*, L 70/1.

[CR12] EC, European Commission, (2006). Regulation No 1881/2006 of 19 December 2006 setting maximum levels for certain contaminants in foodstuffs*, Official Journal of the European Union***,** L 364/5.

[CR13] EC, European Commission, (2011). Regulation No 835/2011 of 19 August 2011 amending Regulation (EC) No 1881/2006 as regards maximum levels for polycyclic aromatic hydrocarbons in foodstuffs. *Official Journal of the European Union***,** L 215/4.

[CR14] EFSA, European Food Safety Authority (2008). Polycyclic aromatic hydrocarbons in food scientific opinion of the panel on contaminants in the food chain. EFSA Journal.

[CR15] FSA, Food Standards Agency, (2012). Polycyclic aromatic hydrocarbons in cereals. Cereal products. Vegetables. Vegetable products and traditionally smoked foods. Food survey information sheet number 01/12 april 2012.

[CR16] Harner T, Shoeib M, Diamond M, Stern G, Rosenberg B (2004). Using passive air samplers to assess urban–rural trends for persistent organic pollutants: poly-chlorinated biphenyls and organochlorine pesticides. Environmental Science and Technology.

[CR17] Hites RK, Day HR (1992). Unusual persistence of DDT in some Western USA soils. Bulletin of Environmental Contamination and Toxicology.

[CR18] Jagadish GK, Jaylakshmi SK, Sreeramulu K (2015). Evaluation of pesticide residue in rice, wheat and pulses of Bidar district Karnataka, India. Issues in Biological Sciences and Pharmaceutical Research.

[CR19] Kipopoulou AM, Manoli E, Samara C (1999). Bioconcentration of polycyclic aromatic hydrocarbons in vegetables grown in an industrial area. Environmental Pollution.

[CR20] Kobayashia R, Okamotob RA, Maddalenac RL, Kado NY (2008). Polycyclic aromatic hydrocarbons in edible grain: a pilot study of agricultural crops as a human exposure pathway for environmental contaminants using wheat as a model crop. Environmental Research.

[CR21] KOWR, Krajowy Ośrodek Wspracia Rolnictwa (2013). Rynek Zbóż w Polsce http://www.kowr.gov.pl/uploads/rynek-zboz-2013-pl.pdf, accessed 13.06.2019.

[CR22] Lawal AT (2017). Polycyclic aromatic hydrocarbons a review. Cogent Environmental Sciences.

[CR23] Lin H, Tao S, Zuo Q, Convey RM (2007). Uptake of polycyclic aromatic hydrocarbons by maize plants. Environmental Pollution.

[CR24] Liu W, Wang Y, Chen Y, Tao S, Liu W (2017). Polycyclic aromatic hydrocarbons in ambient air, surface soil and wheat grain near a large steel-smelting manufacturer in northern China. Journal of Environmental Sciences.

[CR25] Liu, X., Zhang, G., Li, J., Yu L.-L., Xu, Y., Li, X.-D., Kobara, Y., Jones, K.C. (2009). Seasonal Patterns and Current Sources of DDTs, Chlordanes, Hexachlorobenzene, and Endosulfan in the Atmosphere of 37 Chinese Cities. *Environmental Science and Technolology* 43, 1316–1321. 10.1021/es802371n 19350897

[CR26] Łozowicka B, Kaczyński P (2009). Linuron, DDT and organochlorine pesticide residues in plants from North-Eastern Poland. Ecological Chemistry and Engineering A.

[CR27] Magi E, Bianco R, Ianni C, Di Carro M (2002). Distribution of polycyclic aromatic hydrocarbons in the sediments of the Adriatic Sea. Environmental Pollution.

[CR28] Mottier P, Parisod V, Turesky RJ (2000). Quantitative determination of polycyclic aromatic hydrocarbons in barbecued meat sausages by gas chromatography coupled to mass spectrometry. Journal of Agricultural and Food Chemistry.

[CR29] Nfon E, Cousins IT, Broman D (2008). Biomagnification of organic pollutants in benthic and pelagic marine food chains from the Baltic Sea. Science of the Total Environment.

[CR30] Okay OS, Karacık B, Henkelmann B, Schramm KW (2011). Distribution of organochlorine pesticides in sediments and mussels from the Istanbul strait. Environmental Monitoring and Assessment.

[CR31] Pampanin, D.M., Sydnes, M.O., (2017). Petrogenic *polycyclic aromatic hydrocarbons in the aquatic environment*: analysis, synthesis, toxicity and environmental impact. Bentham eBooks.

[CR32] Paris A, Ledauphin J, Poinot P, Gaillard JL (2018). Polycyclic aromatic hydrocarbons in fruits and vegetables: origin, analysis, and occurrence. Environmental Pollution.

[CR33] Phillips DH (1999). Polycyclic aromatic hydrocarbons in the diet. Mutation Research.

[CR34] Pinsuwan S, Li L, Yalkowsky SH (1995). Correlation of octanol/water solubility ratios and partition coefficients. Journal of Chemical and Engineering Data.

[CR35] Qiao M, Wang C, Huang S, Wang D, Wang Z (2006). Composition, sources, and potential toxicological significance of PAHs in the surface sediments of the Meiliang Bay, Taihu Lake, China. Environment International.

[CR36] Qiu X, Zhu T, Yao B, Hu J, Hu S (2005). Contribution of dicofol to the current DDT pollution in China. Environmental Science and Technology.

[CR37] Roszko M, Szymczyk K (2009). Determination of pesticides in selected cereal products by gas chromatography and ion trap mass spectrometry. Chemia Analityczna.

[CR38] Roszko M, Jedrzejczak R, Szymczyk K (2014). Polychlorinated biphenyls (PCBs), polychlorinated diphenyl ethers (PBDEs) and organochlorine pesticides in selected cereals available on the Polish retail market. Science of the Total Environment.

[CR39] Roszko ML, Kamińska M, Szymczyk K, Jȩdrzejczak R (2016). Levels of selected persistent organic pollutants (PCB, PBDE) and pesticides in honey bee pollen sampled in Poland. PLoS One.

[CR40] Roszko M, Kaminska M, Szymczy KK, Jędrzejczak R (2018). Dietary risk evaluation for 28 polycyclic aromatic hydrocarbons (PAHs) in tea preparations made of teas available on the polish retail market. Environmental Science and Health B.

[CR41] Rugen PJ, Stem CD, Lamm SH (1989). Comparative carcinogenicity of the PAHs as a basis for acceptable exposure levels (AELs) in drinking water. Regulatory Toxicology and Pharmacology.

[CR42] Saclo HH, Garrigues P, Ewald M (2000). Origin of polycyclic aromatic hydrocarbons (PAHs) in coastal marine sediments: case studies in Cotonou (Benin) and Aquitaine (France) areas. Marine Pollution Bulletin.

[CR43] Santos MM, Brehm FA, Filippe TC, Reichert G, Azevedo JCR (2017). PAHs diagnostic ratios for the distinction of petrogenic and pirogenic sources: applicability in the Upper Iguassu Watershed - Parana, Brazil. Brazilian Journal of Water Resources.

[CR44] Tao S, Li XR, Yang Y, Coveney RM (2006). Dispersion modeling of polycyclic aromatic hydrocarbons from combustion of biomass and fossil fuels and production of coke in Tianjin, China. Environmental Science and Technology.

[CR45] Tobiszewski M, Namiesnik J (2012). PAH diagnostic ratios for the identification of pollution emission sources. Environmental Pollution.

[CR46] Toteja GS, Diwakar S, Mukherjee A, Singh P, Saxena BN, Kalra RL, Kapoor SK, Kaur H, Raizada RB, Singh V, Vaidya RC, Chakraborty S, Shirolkar SB, Regupathy A, Douressamy (2006). Residues of DDT and HCH in wheat samples collected from different states of India and their dietary exposure: a multicentre study. Food Additives and Contaminants.

[CR47] UN, United Nations, (2007). Stockholm Convention on persistent organic pollutants (POPs).

[CR48] Vromman V, Maghuin-Rogister G, Vleminckx C, Saegerman C, Pussemier L, Huyghebaert A (2014). Risk ranking priority of carcinogenic and/or genotoxic environmental contaminants in food in Belgium. Food Additives & Contaminants, Part A: Chemistry, Analysis, Control, Exposure & Risk Assessment.

[CR49] Wang XP, Xu BQ, Kang SC, Cong ZY, Yao TD (2008). The historical residue trends of DDT, hexachlorocyclohexanes and polycyclic aromatic hydrocarbons in an ice core from Mt. Everest, Central Himalayas, China. Atmospheric Environment.

[CR50] Yohannes YB, Ikenaka Y, Saengtienchai A, Watanabe KP, Nakayama SMM, Ishizuka M (2013). Occurrence, distribution, and ecological risk assessment of DDTs and heavy metals in surface sediments from Lake Awassa—Ethiopian Rift Valley Lake. Environmental Science and Pollution Research.

[CR51] Yunker MB, Macdonald RW, Vingarzan R, Mitchell RH, Goyette D, Sylvestre S (2002). PAHs in the Fraser River basin: a critical appraisal of PAH ratios as indicators of PAH source and composition. Organic Geochemistry.

[CR52] Yunker MB, Macdonald RW, Snowdon LR, Fowler BR (2011). Alkane and PAH biomarkers as tracers of terrigenous organic carbon, in Arctic Ocean sediments. Organic Geochemistry.

[CR53] Zhang J, Cai L, Yuan D, Chen M (2004). Distribution and sources of polynuclear aromatic hydrocarbons in mangrove surficial sediments of Deep Bay, China. Marine Pollution Bulletin.

[CR54] Zhang LF, Dong L, Shi SX, Zhou L, Zhang T, Huang YR (2009). Organochlorine pesticides contamination in surface soils from two pesticide factories in Southeast China. Chemosphere.

[CR55] Zhang C, Liu L, Ma Y, Li F (2018). Using isomeric and metabolic ratios of DDT to identify the sources and fate of DDT in Chinese agricultural topsoil. Environmental Science and Technology.

[CR56] Ziegenhals K, Jira W, Speer K (2008). Polycyclic aromatic hydrocarbons (PAH) in various types of tea. European Food Research and Technology.

